# Vorwort der Herausgeber

**DOI:** 10.1007/s11943-021-00300-4

**Published:** 2021-12-17

**Authors:** Timo Schmid, Markus Zwick

**Affiliations:** Institut für Forschung und Entwicklung in der Bundesstatistik, Statistisches Bundesamt, Gustav-Stresemann-Ring 11, 65189 Wiesbaden, Deutschland

Liebe Leserinnen und Leser,

rechtzeitig vor Weihnachten erscheint auch dieses Jahr wieder die diesjährig letzte Ausgabe des AStA (Wirtschafts- und Sozialstatistisches Archiv). Rückblickend ist dieses Jahr von Wandel und Anpassung geprägt und doch erleben wir ein gefühltes Déjà-vu aufgrund der weitreichenden Auswirkungen der andauernden Pandemie. Auch 2021 waren die täglichen Nachrichten wieder zunehmend von deskriptiven Grafiken und Prognosen, also letztendlich statistischen Verfahren, geprägt. Nicht zuletzt waren auch die zahlreichen Wahlen in Deutschland ein Grund hierfür. Empirische Evidenz fordert aber stets eine transparente Diskussion und Weiterentwicklung von statistischen Methoden. Es freut uns daher als Plattform unseren Beitrag zu leisten, der nur mit Ihnen als Verfasser und Verfasserinnen von wissenschaftlichen Texten und als Leser und Leserinnen funktioniert.

Rendtel et al. (2021a) eröffnen diese Ausgabe mit ihrem Beitrag: *„Die Erforschung der Dynamik der Corona-Pandemie in Deutschland: Survey-Konzepte und eine exemplarische Umsetzung mit dem Sozio-oekonomischen Panel (SOEP).“ *Im Rahmen der durch die Weltgesundheitsorganisation (WHO) veröffentlichten Richtlinien für Bevölkerungsstichproben aus dem Frühjahr 2020 diskutieren Rendtel et al. (2021a) verschiedene Möglichkeiten und praktischen Grenzen der Survey-Statistik. Ein zentraler Aspekt ist die Zusammenführung medizinischer Tests mit bevölkerungsrepräsentativen Umfragen, wobei sich gerade bei personalisierten Rückmeldungen von Testergebnissen ein Spannungsfeld hinsichtlich des Datenschutzes eröffnet. Während sich der erste Teil des Textes mit Survey-statistischer Methodologie befasst, diskutieren Rendtel et al. (2021a) im zweiten Teil die Nutzung des SOEP für eine WHO-konforme Covid-19-Erhebung. Es werden erste Ergebnisse aus dem Projekt einer Kooperation des Robert Koch-Instituts (RKI) mit dem SOEP diskutiert.

Im Rahmen des Beitrags „Pendler Mobil: Die Verwendung von Mobilfunkdaten zur Unterstützung der amtlichen Pendlerstatistik“ untersucht Hadam (2021), ob und wie die amtliche Pendlerrechnung mit Mobilfunkdaten unterstützt werden kann. Am Beispiel des Bundeslandes Nordrhein-Westfalen vergleicht sie Mobilfunkdaten aus dem Netz der Deutschen Telekom mit Mobilitätsdaten der amtlichen Pendlerrechnung. Mobilfunkdaten bieten aufgrund ihrer zeitlichen Aktualität und kleinräumigen Auflösung das Potenzial Pendlerbewegungen, unterstützt durch die etablierte Pendlerrechnung, zu schätzen. Hadam (2021) definiert plausible Kriterien, um Pendlerbewegungen aus den Mobilfunkdaten herauszufiltern und stellt eine hohe Korrelation der so gewonnenen Mobilitätsvariablen und denen der amtlichen Pendlerrechnung fest. Herausforderungen einer Unterstützung der Pendlerrechnung mit Mobilfunkdaten manifestieren sich in den Über- oder Unterschätzungen der Pendlerverflechtungen durch inadäquate Filterkriterien sowie im Aufbereitungsprozess der Mobilfunkdaten.

Ein ebenso dauerhaft präsentes Thema, das gesundheits- und umweltpolitische Aspekte vereint, findet sich im Beitrag „Feinstaubbelastung und Lebenserwartung in Deutschland“ von Prinz und Richter (2021). Hierfür werden primär Daten von Feinstaubmessstationen der Jahre 2002–2016 verwendet. Als Hilfsinformationen der Lebenserwartung werden auf Kreisebene verfügbare Einkommen pro Kopf sowie weitere sozio-demographische Variablen und ein nichtlinearer Zeittrend in die Untersuchung einbezogen. Prinz und Richter (2021) finden einen negativen Zusammenhang zwischen Feinstaubbelastung und Lebenserwartung, verorten jedoch Unterschiede zwischen geographisch westlichen und östlichen Teilen Deutschlands.

Mit ihrem Beitrag „Emil Julius Gumbel – Innovativer Statistiker und engagierter Pazifist“ würdigen Rendtel et al. (2021b) den Namensgeber der jährlichen Gumbel-Vorlesung auf der Statistischen Woche. Dieses Portrait beschreibt den Lebensweg eines Forschers, dessen Leistungen weit über das wissenschaftliche Wirken hinausreichen. Vom Weltkriegsteilnehmer zum Pazifisten, der politische Morde in der frühen Weimarer Republik auf Basis statistischer Schlüsse analysierte und sich als Statistik-Professor an der Universität Heidelberg verschiedenen deutsch-nationalideologischen Angriffen ausgesetzt sah. Rendtel et al. (2021b) schildern Gumbels Emigration über Frankreich in die USA und seine vergeblichen Versuche, wieder an deutschen Universitäten aufgenommen zu werden. Neben persönlichen Anekdoten eines früheren Wegbegleiters, findet sich in diesem Beitrag nicht zuletzt auch eine kurze Zusammenfassung des wissenschaftlichen Werks im Bereich der Statistik der Extremwerte.

Für das Interview dieser Ausgabe konnte Walter Krämer (2021) einen der prägenden deutschen Wissenschaftler in der Schnittmenge zwischen Volkswirtschaftslehre und Statistik, Gerd Hansen, gewinnen. Sein Lebenslauf spiegelt seine Verbundenheit zu beiden Fachrichtungen. Gerd Hansen studierte Volkswirtschaftslehre an der Christian-Albrechts-Universität zu Kiel, um im Anschluss bei Heinz Gollnick am Institut für Statistik und Ökonometrie in Hamburg zu promovieren. Bis zu seiner Emeritierung 2003 wirkte Gerd Hansen als Professor für Ökonometrie an der Universität Kiel. Er war Mitherausgeber des Allgemeinen Statistischen Archivs (1973–1980) sowie Vorsitzender des Ausschusses für Empirische Wirtschaftsforschung und Angewandte Ökonometrie (1982–1986). Im Interview spricht der Ökonometriker über die wichtigsten Entwicklungen des Fachs wie zum Beispiel die vermehrte Anwendung von nichtlinearen Verfahren und den Umgang mit der Verfügbarkeit von großen Datenmengen.

Wie immer bedanken wir uns an dieser Stelle wieder für alle Einreichungen bei AStA, dem Wirtschafts- und Sozialstatistischen Archiv. Insbesondere gilt unser Dank den Autoren und Autorinnen für Ihre innovativen Beiträge. Den Mitgliedern des Beirats der Zeitschrift und den zahlreichen Reviewern und Reviewerinnen danken wir ebenso, denn ihre großartige und ehrenamtliche Arbeit trägt maßgeblich zur Sicherung der Qualität des AStA bei. Ihnen, liebe Leserinnen und Leser, wollen wir für Ihre Treue und Ihr Interesse danken und hoffen, Sie mit den bisherigen Ausgaben auch für eigene Beiträge in künftigen Ausgaben zu inspirieren. Unterstützen Sie uns beispielsweise neben wissenschaftlichen Einreichungen gerne auch mit Vorschlägen für Buchbesprechungen, herausragenden Masterarbeiten zur Veröffentlichung in Kurzform, Vorstellungen von bedeutenden Entwicklungen auf statistischen Lehrstühlen oder in statistischen Ämtern.

Abschließend wünschen wir Ihnen und Ihren Familien und Freunden ein frohes Weihnachtsfest, andauernde Gesundheit sowie alles Gute für das Jahr 2022.

Timo Schmid und Markus Zwick
